# A capillary-based microfluidic device enables primary high-throughput room-temperature crystallographic screening

**DOI:** 10.1107/S1600576721004155

**Published:** 2021-06-14

**Authors:** Shuo Sui, Anne Mulichak, Raviraj Kulathila, Joshua McGee, Danny Filiatreault, Sarthak Saha, Aina Cohen, Jinhu Song, Holly Hung, Jonathan Selway, Christina Kirby, Om K. Shrestha, Wilhelm Weihofen, Michelle Fodor, Mei Xu, Rajiv Chopra, Sarah L. Perry

**Affiliations:** a Department of Chemical Engineering, University of Massachusetts Amherst, Amherst, MA, USA; b IMCA-CAT, Argonne National Laboratory, Lemont, IL, USA; c Novartis Institutes for BioMedical Research, Cambridge, MA, USA; d Macromolecular Crystallography Group, Stanford Synchrotron Radiation Lightsource, Menlo Park, CA, USA; e SPT Labtech Ltd, Boston, MA, USA; f Department of Biochemistry and Molecular Biology, University of Massachusetts Amherst, Amherst, MA, USA

**Keywords:** compound screening, microfluidics, structural biology, protein crystallography, X-ray diffraction

## Abstract

A novel capillary-based microfluidic strategy to accelerate the process of small-molecule-compound screening by room-temperature X-ray crystallography using protein crystals is reported. The ultra-thin microfluidic devices are composed of a UV-curable polymer, patterned by cleanroom photolithography, and have nine capillary channels per chip. The chip was designed for ease of sample manipulation, sample stability and minimal X-ray background. 3D-printed frames and cassettes conforming to SBS standards are used to house the capillary chips, providing additional mechanical stability and compatibility with automated liquid- and sample-handling robotics. These devices enable an innovative *in situ* crystal-soaking screening workflow, akin to high-throughput compound screening, such that quantitative electron density maps sufficient to determine weak binding events are efficiently obtained. This work paves the way for adopting a room-temperature microfluidics-based sample delivery method at synchrotron sources to facilitate high-throughput protein-crystallography-based screening of compounds at high concentration with the aim of discovering novel binding events in an automated manner.

## Abstract 2

A novel capillary-based microfluidic strategy to accelerate the process of small-molecule-compound screening by room-temperature X-ray crystallography using protein crystals is reported.

## 1. Introduction   

Modern target-based drug discovery utilizes biochemical and cellular assay screening of small-molecule chemical libraries, containing millions of compounds, in primary high-throughput screens (HTS) that are conducted at a single small-molecule concentration with a binary readout of no hit/hit (Bleicher *et al.*, 2003 ▸; Walters & Namchuk, 2003 ▸; Hughes *et al.*, 2011 ▸). Following this, secondary assays, including dose-response measurements, are conducted to fully characterize and prioritize hits and initiate lead-discovery medicinal chemistry efforts (Geysen *et al.*, 2003 ▸). The full structural characterization of small-molecule hits from HTS using X-ray crystallography is routinely applied for multiple impacts including validation, prioritization, elucidating mechanisms of actions and structure-guided drug design. X-ray crystallography is also remarkably sensitive to weak binding events, but its utility as a primary screening technique is constrained by both the need to obtain crystals of target proteins and the manual burden of conventional single-crystal sample handling. Several pioneering reports have explored X-ray-crystallography-based compound-screening processes (Chilingaryan *et al.*, 2012 ▸; Collins *et al.*, 2017 ▸; Yin *et al.*, 2014 ▸). The work presented here is an effort to create a new device to perform room-temperature primary HTS with X-ray crystallography, prior to the structural characterization of small-molecule-compound hits.

Co-crystallization and soaking are two established techniques to form protein–small-molecule complexes, where small-molecule compounds are added either before or after the protein is crystallized, respectively (Hassell *et al.*, 2007 ▸). Soaking of crystals with high concentrations of small-molecule compounds has been effective at discovering weak interactions and enabling fragment-based drug discovery (Nienaber *et al.*, 2000 ▸; Blundell *et al.*, 2002 ▸). Because the crystal manipulations are time and labor intensive and often introduce physical damage to fragile protein crystals, several elegant robotic solutions have been developed to minimize manual steps and are in the process of being widely adopted (Wright *et al.*, 2019 ▸; Samara *et al.*, 2018 ▸).

The efficiency of X-ray crystallography data collection has also been the focus of extensive improvements in synchrotron data collection (Helliwell & Mitchell, 2015 ▸). Modern protein crystallography beamlines utilize raster scanning to center crystals with high-intensity collimated X-ray beams and, combined with high-speed X-ray detectors, enable full data sets to be collected in seconds (Russi *et al.*, 2016 ▸; Cherezov *et al.*, 2009 ▸; Papp *et al.*, 2017 ▸). Currently, sample delivery to the beam accounts for a significant part of the cycle time, and these delivery challenges at synchrotron beamlines have been addressed with automated robotics and by redesigning sample holders to accommodate many crystals in a single mount (Roedig *et al.*, 2015 ▸, 2016 ▸, 2017 ▸; Bingel-Erlenmeyer *et al.*, 2011 ▸; Heymann *et al.*, 2014 ▸; Coquelle *et al.*, 2015 ▸; Feld *et al.*, 2015 ▸; Maire *et al.*, 2011 ▸; Axford *et al.*, 2012 ▸; Baxter *et al.*, 2016 ▸; Mueller *et al.*, 2015 ▸; Oghbaey *et al.*, 2016 ▸; Doak *et al.*, 2018 ▸; Zarrine-Afsar *et al.*, 2012 ▸; Jacquamet *et al.*, 2004 ▸).

Microfluidic platforms are a new sample-delivery strategy that has shown great potential for enabling high-throughput X-ray crystallography experiments (Sui & Perry, 2017 ▸; Martiel *et al.*, 2019 ▸). One major advantage of employing microfluidics is the ability to perform *in situ* data collection, minimizing the need for manual crystal manipulations (Roedig *et al.*, 2015 ▸; Schieferstein *et al.*, 2017 ▸; Pawate *et al.*, 2015 ▸; Martiel *et al.*, 2019 ▸; Heymann *et al.*, 2014 ▸; Sui & Perry, 2017 ▸; Abdallah *et al.*, 2015 ▸; Dhouib *et al.*, 2009 ▸; Perry *et al.*, 2013 ▸, 2014 ▸; Pinker *et al.*, 2013 ▸; Zarrine-Afsar *et al.*, 2012 ▸; Guha *et al.*, 2012 ▸; Mueller *et al.*, 2015 ▸; Sauter *et al.*, 2007 ▸; Stojanoff *et al.*, 2011 ▸; Baxter *et al.*, 2016 ▸). We set out to create a microfluidics-based X-ray crystallography screening workflow that enables the soaking of crystals with high concentrations of small-molecule compounds, automatable liquid handling of all reagents which potentially include protein crystals and stable sample shipping, and is compatible with existing synchrotron beamline infrastructure to collect diffraction data of sufficient quality. We define the last criterion as being sufficient to enable the calculation of electron density maps that qualitatively identify binding events. The discovered small-molecule-compound hits are fully characterized using single-crystal structure determination.

Here we introduce a novel monolithic photoresist-based microfluidic platform that uses a unique three-layer assembly process to create an array of capillary channels such that the thickness of the material present along the X-ray beam path is less than 40 µm. The device layout was designed and precisely fabricated to be compatible with standardized well-plate layouts and, coupled with 3D-printed support frames and cassettes, to facilitate liquid handling and beamline automation. Device loading takes advantage of capillary forces to facilitate reagent manipulation. The device was designed to allow small-molecule compounds to coat the channels prior to the addition of crystals for *in situ* crystal soaking followed by *in situ* X-ray diffraction data collection. We have tested our approach with multiple protein crystal systems, with data obtained from trypsin and galectin-3 (Gal-3) presented here, including a compound screen and a dose-response study, and have demonstrated compatibility with three beamlines at two different synchrotrons. As a result, we have improved sample preparation and data collection throughput and obtained high-quality structural information reading out the presence of small-molecule compounds to enable an automated workflow for the screening of small-molecule compounds by X-ray crystallography.

## 2. Methods   

### 2.1. Photomask design   

**Figure 1:**
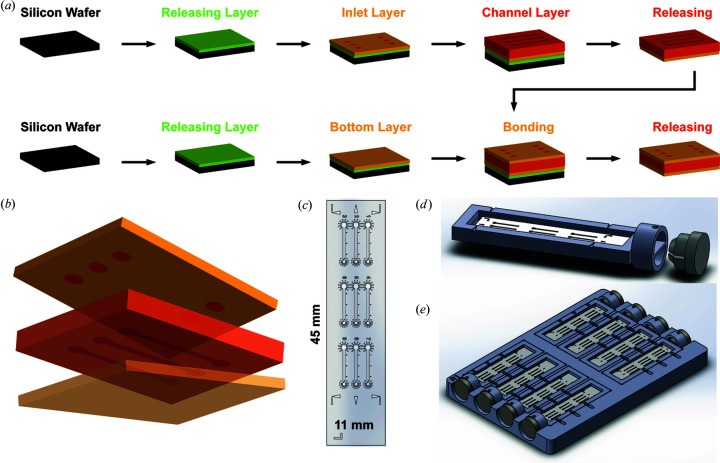
(*a*) An overview of the device fabrication flow, illustrating the sequence of the major steps of spin coating, bonding and releasing. (*b*) A top view of a device showing its features and layout. (*c*) A simplified 3D view of the device architecture with a top inlet layer, a middle channel layer and a bottom supporting layer. (*d*) A device inserted into a 3D-printed frame connected to a magnetic base, and (*e*) multiple device-inserted frames accommodated in a 3D-printed cassette.

The device features were designed in Adobe *Illustrator*. The chip dimensions are 45 mm long by 11 mm wide. The thickness of the chip and the various features were defined via spin coating. Each chip has nine 6.75 mm-long and 0.4 mm-wide parallel channels arranged in a three by three array. Each channel has one 1 mm-diameter inlet and one 0.6 mm-diameter outlet. The distance between inlets of two adjacent parallel channels was set to 2.25 mm and that of two colinear channels was 11.25 mm, corresponding to intervals of the 2.25 mm well-center spacing of a standard 1536-well plate. There are also two 0.1 mm by 0.2 mm notches near the outlet end of the channel to prevent crystals from exiting the channel during sample filling. Twenty identical 0.16 mm-diameter dots were distributed in a radial manner around the inlet and outlet to reduce the potential for spreading of aqueous solution during pipetting and promote epoxy adhesion for device sealing. Fiducial markers and numberings were added for reference during chip manipulations (Fig. 1 ▸). A film photomask of the design was printed by Fineline Imaging at a resolution of 10 160 d.p.i.

### 2.2. Microfluidic device fabrication   

All materials, unless otherwise described, were used as received. Negative-tone photoresists SU-8 2007, SU-8 2015 and SU-8 2100 and the sacrificial layer LOR 1 A were purchased from Kayaku Advanced Materials (formerly Microchem, Westborough, MA, USA) and kept in a cleanroom environment, away from UV light. Developer solution for SU-8 (propylene glycol methyl ether acetate, PGMEA) was purchased from Sigma–Aldrich. RD-6 developer for the LOR 1A (tetramethylammonium hydroxide) was purchased from Futurrex (Franklin, NJ, USA). Silicon wafers [3 inch (76.2 mm), Type P, test grade, 100 orientations; University Wafer, Boston, MA, USA] were cleaned with acetone (ACS reagent, Fisher Scientific) and dried with nitrogen.

The inlet/channel layer of the device was prepared using the following procedure. LOR 1A (2 ml) was dispensed onto the silicon wafer and spin-coated at 4000 r min^−1^ for 60 s with a 5 s ramp (Brewer Science CEE 100CB), and subsequently baked at 458 K for 5 min to create the 100 nm-thick release layer. SU-8 2015 (2 ml) was then dispensed and spin-coated on the LOR layer at 4000 r min^−1^ for 60 s with a 5 s ramp, with a subsequent soft bake at 368 K for 3 min. The resulting film thickness was approximately 12 µm. This photoresist layer was patterned with the inlet layer photomask by standard photolithography with a Mask Aligner (SUSS MA/BA 6, UV lamp power 9.5 mW cm^−2^) using a 12 s exposure. After a 3 min post-exposure bake at 368 K, the sample was allowed to cool. SU-8 2100 (2 ml) was then dispensed and spin-coated onto the inlet layer at a rate of 2000 r min^−1^ for 60 s with a 5 s ramp, giving a film thickness of around 140 µm. A two-stage soft bake was used for this layer, starting with 5 min at 338 K, followed by 30 min at 368 K. Then the features of the channel layer were patterned using a 26 s UV exposure after the channel photomask was aligned with the inlet layers. A post-exposure bake was subsequently performed at 338 K for 5 min and 368 K for 12 min. After cooling in air, the SU-8 layers were developed by PGMEA to reveal the channel and inlet structures. The entire wafer was then dried using a nitrogen flow and was allowed to develop for at least 8 h in RD-6 solution to dissolve the sacrificial LOR 1A release layer. The final free-standing structure was rinsed with deionized (DI) water (Millipore) and air-dried.

A second wafer was cleaned and coated with LOR 1A and SU-8 2015 as described above to form the bottom layer of the device. After processing, a second layer of SU-8 2015 was spin-coated above with the same procedure but soft baked for only 5 min to serve as an adhesion layer. The total thickness of the bottom layer was 24 µm. The previously released inlet/channel layer was then visually aligned and bonded to the bottom layer using a nanoimprinter (Nanonex, Monmouth Junction, NJ, USA) at 341 K and 10 psi ( 68.95 kPa) for 3 min. The resulting structure was further baked at 368 K for 3 min to achieve a solid layer contact and cooled at a rate of 3 K min^−1^ to minimize stress build up due to differences in the thermal expansion of the SU-8 layers and the substrate. The assembled device was consecutively aligned with the photomask for the bottom layer, exposed to UV light for 12 s, baked at 368 K for 3 min and cooled at 3 K min^−1^. After PGMEA development to remove uncured photoresist, the assembled device was released from the wafer surface by developing in RD-6 for at least 8 h. The final device was rinsed in DI water and air-dried.

### 2.3. 3D-printed frame and cassette   

All 3D-printed pieces were designed using *SolidWorks 2018–2019* (https://www.solidworks.com/) and subsequently printed using an Objet Connex 350 printer with VeroClear, VeroBlack and VeroWhite photopolymer resins. A frame was designed in order to enhance the stability of the chip and to provide compatibility with a modified magnetic base (Crystal Positioning Systems, Jamestown, NY, USA) for mounting at the synchrotron [Fig. 1 ▸(*c*)]. The dimensions of the frame were 16 × 51 × 2.5 mm. The bottom of the mounting frame contained a 13 mm circular extrusion for connection to the magnetic base, which was secured through the tightening of a set-screw on the magnetic base assembly. The chip was inserted into and stabilized by a 420 µm groove. Fiducial marks were located along the mounting frame and the microfluidic chip to assist in loading and mounting efforts.

A cassette was designed to secure and organize the microfluidic chips for sample handling. The cassette was designed to be compatible (85.48 × 127.76 mm) with standard well-plate handling equipment and could hold eight microfluidic chips mounted on frames. To assist in imaging of the loaded chips, the top and bottom of the cassette were open. Mounting frames loaded with microfluidic chips were horizontally inserted into the shipping cassette. To ensure that the loaded frames remain securely inside of the cassette, a slide lock could be inserted once loaded.

To automate the process of filling each channel, the layout of the channels within the larger cassette assembly was designed using the footprint of a 1536-well plate. The filling cassette was designed be compatible with a mosquito liquid-handling robot (SPT Labtech). To account for the depression of the eight mosquito tips simultaneously, indentations were added to each side of the frame inserts and the cassette foundation. Specialized frames were designed with a similar style to the mounting frame, with the addition of the holes to accommodate the dispensing tips.

### 2.4. Device characterization   

**Figure 2:**
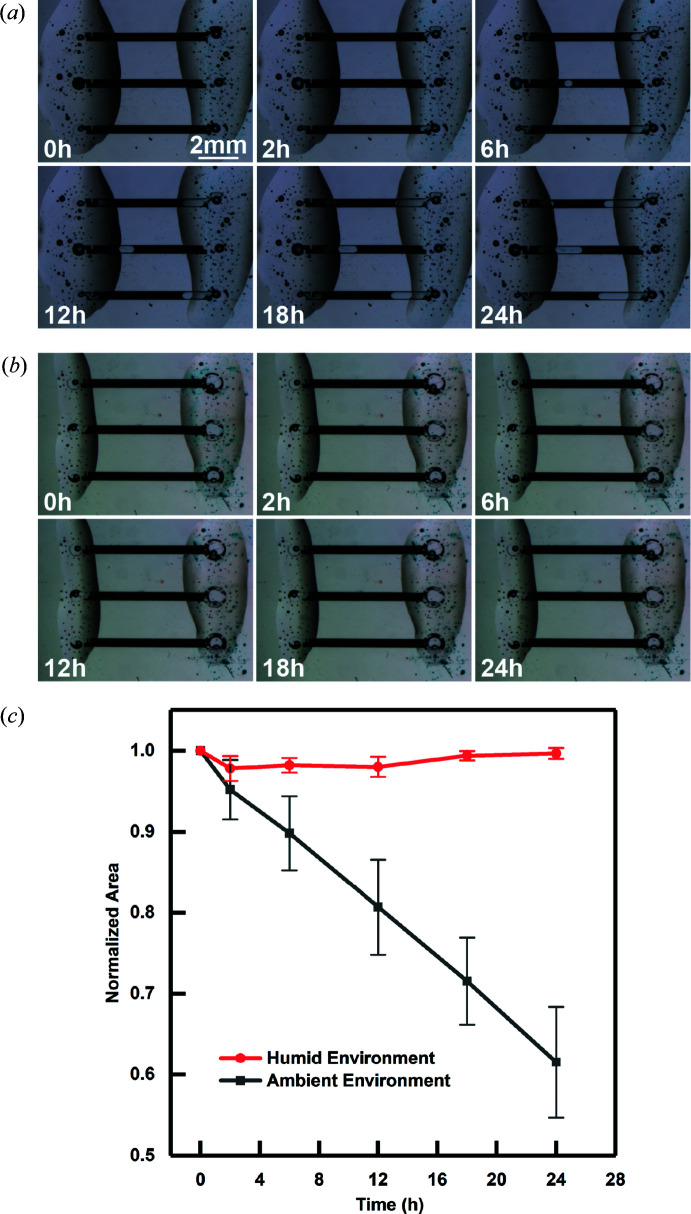
Optical micrographs of dye-filled and epoxy-sealed channels showing changes of content volume over time (*a*) in an ambient environment and (*b*) in a humid environment with a water-saturated Kimwipe tissue in a sealed Petri dish. (*c*) Measurement of dye-occupied area as a function of time in both humid and ambient environments. Data were normalized to the 0 h measurement and the average was calculated from measurements from three channels.

The film thicknesses were determined by releasing spin-coated SU-8 films from the substrate and measuring three times with a micrometer (Mitutoyo). Two sets of evaporation tests were performed using aqueous solutions of food coloring (McCormick) for visualization (Fig. 2 ▸). The solution was pipetted onto the inlets and flowed into the channels by capillary action. The channels were then sealed with epoxy. The first experiment monitored the loss of water from channels exposed to the ambient environment. The second experiment considered the effect of a humidified environment by placing the chip into a sealed Petri dish (Fisher Scientific) alongside wet Kimwipe tissues. Each experiment was performed in triplicate, considering three channels on the same device. Evaporation was monitored over time using a Zeiss SteREO microscope, and water loss was determined by tracking the solution occupancy area changes quantified by *ImageJ* (Schneider *et al.*, 2012 ▸) over a period of 24 h.

**Figure 3:**
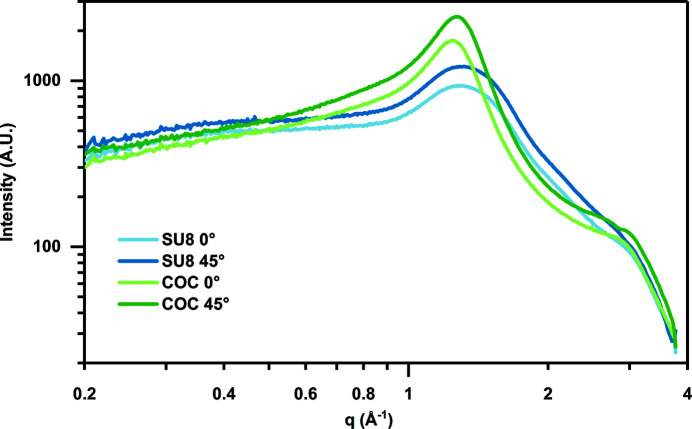
One-dimensional integrated X-ray intensity as a function of scattering vector magnitude *q*, showing background scattering of 150 µm-thick SU-8 (blue) and COC (green) films placed either at 0° (perpendicular to the incident beam) or at an angle of 45°.

An X-ray generator (Rigaku Xtalab PRO MM007) with an X-ray wavelength of 1.54 Å and a beam size of 200 µm was used to investigate the background scatter of our materials. A 150 µm-thick free-standing SU-8 photoresist film was mounted on a customized magnetic base (Crystal Positioning Systems) perpendicular to the X-ray beam path. A 150 µm-thick film of cyclic olefin copolymer (TOPAS 6013) was mounted the same way. The distance between the film and the detector (PILATUS3 R 200K) was set to be 40 mm, giving a maximum resolution of 1.96 Å. Data collection was performed at sample orientations of 0 and 45° to compare the effect of material thickness (Fig. 3 ▸). X-ray snapshots were collected using a 10 s exposure and a 0.25° frame width. Background scattering was analyzed using the *Nika* software for 2D data reduction via integration in 2θ (Ilavsky, 2012 ▸).

### 2.5. Trypsin crystallization   

A 60 mg ml^−1^ sample of bovine pancreas trypsin (Sigma T7309) was prepared in 10 mg ml^−1^ benzamidine (VWR-Amresco Life Science), 3 m*M* calcium chloride and 20 m*M* Tris buffer at pH 8.0. Room-temperature crystals of various size and number were grown by varying 15–40%(*w*/*v*) PEG 4000 in 5% increments and 20–35%(*w*/*v*) ethylene glycol in 5% increments across a 24-well sitting-drop tray (HR3-160-Cryschem plate). After addition of 0.2 *M* Li_2_SO_4_ and 0.1 *M* 2-(*N*-morpholino)ethanesulfonic acid at pH 6.5 to each well, the total well buffer volume was brought to 1.0 ml with deionized water. Once the crystals grew, 10 µl of the well buffer from the next well to the right (3–5% higher concentration of PEG 4000) was added and mixed gently without breaking the crystals to ensure that the crystals were not stuck to the plastic. Crystals of Gal-3 protein in complex with a FAb (unpublished) were obtained using 10 mg ml^−1^ of the protein in buffer with 20 m*M* 4-(2-hydroxyethyl)-1-piperazine­ethane­sulfonic acid (HEPES) pH 7.5, 50 m*M* NaCl, 1 mg ml^−1^ collagenase utilizing a hanging-drop method with equal-volume addition of the well solution containing 0.1 m*M* HEPES pH 7.5, 20% PEG 4000 and 10% 2-propanol.

### 2.6. Device operation   

**Figure 4:**
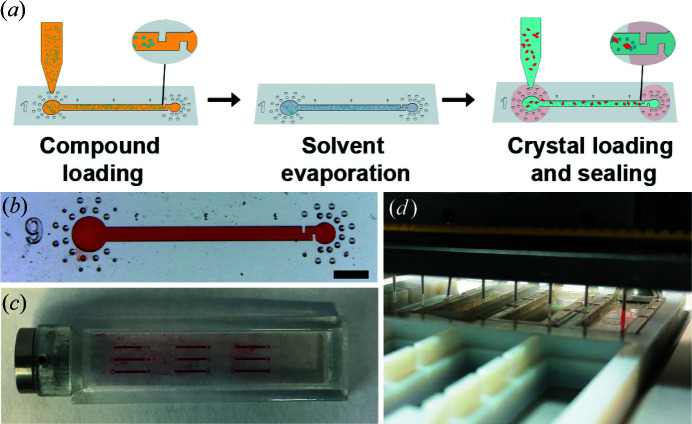
(*a*) Illustration of the compound-screening workflow. Compound molecules were dissolved in DMSO and introduced into the channel by capillary action. The solvent was then allowed to evaporate at room temperature, leaving the compounds coated onto the inner surface of the channel. Finally, a slurry of crystals was introduced into the coated channel by capillary action. Crystals were prevented from passing out of the channel by notches at the outlet end (see inset). The inlets and outlets were then sealed with epoxy. (*b*) Optical micrograph of a channel in a fabricated chip, showing feature details. The channel was filled with red food coloring for visualization. The scale bar represents 1 mm. (*c*) A top view of a fabricated device with all channels filled with red food coloring. The chip was inserted into a 3D-printed frame, which was mounted on a magnetic base. (*d*) A photograph showing the automated sample filling process performed by a mosquito liquid-handling robot. The chip-inserted frame (on the right side) was placed in a 3D-printed cassette having the same size as a standard well plate. The robot was programmed for automated and precise dispensing of solution (red food coloring shown as an example) onto the channel inlets where capillary action was then used to fill the channel.

Approximately 0.5 µl of 10 m*M* compound dissolved in dimethyl sulfoxide (DMSO) was pipetted on top of the inlet of the channel capillary, which allowed the solution to fill the entire channel by capillary action. The chips filled with compound solutions were incubated at room temperature for at least 48 h to allow the DMSO to evaporate. Once the compound was coated on the channel surface and dry, we pipetted up 2 µl of crystal slurry harvested from a sitting-drop tray and sequentially placed less than 0.5 µl on top of multiple channel inlets across the chip. After crystal loading via capillary action, the chip was inserted into a 3D-printed frame. Both channel openings were then sealed by Loctite Instant Mix epoxy adhesive (Fig. 4 ▸).

For shipping of individual chips, the prepared device was placed in a Petri dish (Fisher Scientific) and secured by Scotch tape. A wet tissue was placed near the chip to create a humid environment, and then the Petri dish was sealed by Parafilm (PARAFILM M). Alternatively, a 3D-printed cassette was used to accommodate eight chips inserted into frames. Wet tissues were placed inside the cassette and the entire structure was sealed with well-plate cover tape (Fisher Scientific). Samples were then shipped via overnight courier service to the synchrotron.

### 2.7. Sample loading via liquid-handling robot   

Device channels were prepared using the mosquito liquid-handling robot (SPT Labtech) to deliver 400 nl to the channel inlets. Each disposable micropipette tip utilizes a stainless-steel piston for low-volume positive-displacement pipetting. A custom script was written (freely available from SPT Labtech) to enable automated sample loading to proceed across the 3D-printed cassette using eight tips at a time. Channel inlets spanning four devices in a vertical column can be loaded at once (see Supplementary Video 1).

### 2.8. X-ray diffraction data collection at APS   

**Figure 5:**
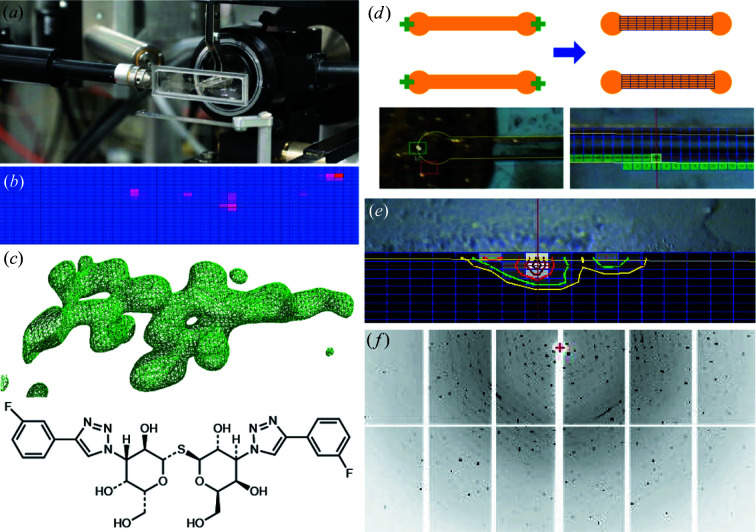
(*a*) Photograph of a device in a 3D-printed frame, mounted on the goniometer via a magnetic base at the IMCA-CAT beamline 17-ID at the APS. (*b*) A heat map obtained from X-ray rastering, showing potential crystal locations. Each square represents a raster area and a deeper red (or higher score) indicates a higher pixel intensity and thus a better chance for finding a strongly diffracting crystal. (*c*) A high-resolution *F*_o_–*F*_c_ electron density map of Gal-3 crystals with TD139 bound based on a crystal identified via raster scanning, along with the molecular structure of the compound. (*d*) An illustration of the procedure for automated sample alignment and raster scanning at SSRL beamlines. A raster grid is overlaid onto the channels after placing anchors at the channel ends to define feature positions. The left photograph shows a channel outline recognized by the software and the right photograph shows the rastering progress. (*e*) Software view of trypsin crystals identified after the raster process with the beam focus targeted onto the crystal. (*f*) An example diffraction pattern from the crystal.

Diffraction data were collected at the IMCA-CAT beamline 17-ID at the Advanced Photon Source (APS) at Argonne National Laboratory. Chips were stored in Styrofoam shipping boxes with moist tissues to maintain humidity and thermal stability prior to data collection. Each chip was manually mounted on the goniometer at the beamline, with the plane of the chip perpendicular to the X-ray beam path (Fig. 5 ▸). To fully access all channels, a translation range of 4.5 mm in the vertical direction and approximately 29 mm along the spindle axis was required. The usable φ rotation range was limited by the proximity of the closest near-sample device, a beam-defining aperture assembly. While the full φ range possible before collision with the aperture assembly varied depending on which row of channels was aligned with the X-ray beam, a conservative range of ±45° was deemed safe for all cases.

Data were collected on a Dectris Pilatus 6M detector that provided continuous shutterless image acquisition with a 12 Hz frame rate. Typically the entire channel or region of interest was rastered for diffraction signal at ±45° to identify crystal locations or the best-diffracting crystals. Low-dose diffraction images were collected along a grid covering the search area and were automatically analyzed for diffraction and scored using the *Spotfinder* software (Zhang *et al.*, 2006 ▸). To minimize the rapid radiation damage observed for samples at room temperature, data sets were collected with a highly attenuated X-ray beam, typically ranging from 0.5 to 2% transmission, depending on the sensitivity of the crystal and the strength of diffraction.

### 2.9. Automated sample rastering at SSRL   

Building on automation previously developed for serial diffraction studies using grid holders (Smith & Cohen, 2008 ▸; Russi *et al.*, 2016 ▸), data collection using the microfluidic device at Stanford Synchrotron Radiation Lightsource (SSRL) started with a semi-automated alignment procedure that utilized a specialized user interface within the *Blu-ice/DCSS* software (McPhillips *et al.*, 2002 ▸). After the device assembly was mounted onto the beamline goniometer, it was rotated edge-on in the inline camera view, and the edge of the grid was clicked within the video display to move it into the X-ray interaction region (and center of rotation of the goniometer). The device was next rotated by 90° to put it face-on in the inline camera view. The circular ends of the top and bottom channels on the edge of the device were clicked in the video display and then the device was translated to put the opposite ends of these channels in the video view. The opposite ends of the top and bottom channels were next clicked to set the grid rotation and orientation. From this information, the location of all the channels was calculated and overlaid on the video view of the device layout [Fig. 5 ▸(*d*) and Supplementary Video 2]. Similar strategies could be carried out for other high-density sample containers of consistent dimensions.

After performing the semi-automated alignment procedure, the software overlaid a grid of positions, representing locations to collect a diffraction pattern, on top of the video image of the channel. Then parameters such as the channel, the beam size and the spacing between diffraction shots were selected and used to collect a grid of low-dose X-ray diffraction images. This grid collection operated as a series of step scans with shutterless collection for each grid point in the row using the Pilatus 6M detector at SSRL BL12-2. The diffraction images collected were automatically analyzed using the program *Spotfinder* (Zhang *et al.*, 2006 ▸), and a profile of the crystal based on the number of diffraction spots in each image was displayed. This enabled the location of crystals or the best diffracting portion of larger crystals to be selected and centered into the X-ray beam for the collection of a complete diffraction data set.

### 2.10. Structure determination   

A custom industrial protein X-ray crystallography data processing and structure determination software pipeline for single crystals (*SnowPipe*; E. Grande, personal communication) was adapted for this project. The data from chip experiments were amenable to this workflow; an HTML-based data-entry software captured the experimental details of the crystal, including protein, compound, crystallographer and data collection parameters. The data were stored in a couchDB format and annotated with shipment date and synchrotron details. Custom Python scripts were then applied to translate anonymized information to synchrotron-compatible data collection input formats. A directory tree was populated with metadata files that described the experiment and enabled automated structure determination with compound fitting utilizing the *GlobalPhasing* (Vonrhein *et al.*, 2011 ▸) software platform and paradigms. Structure refinement was carried out using PDB entry 5h9p as a partial search model (Hsieh *et al.*, 2016 ▸) bound to a proprietary FAb. Finally, the electron density was displayed in a web browser for initial inspection. Multiple data sets from each channel were treated independently and also merged.

## 3. Results   

### 3.1. Microfluidic crystal mounts   

A challenge in designing a microfluidic platform for high-throughput crystallography experiments is the need to balance sample stability with low X-ray background. Traditional cryo-crystallography overcomes both issues by storing samples at liquid-nitrogen temperatures in a cryoprotectant solution, practically eliminating any encasing material (Pflugrath, 2015 ▸). Experiments at room temperature require a different approach (Fraser *et al.*, 2011 ▸). Thicker materials that help to prevent sample dehydration result in increased background noise and signal attenuation during X-ray data collection (Shelby *et al.*, 2020 ▸). Therefore, it is necessary to optimize material selection and thickness. Generally, a thin film of an amorphous material composed of light atoms, such as a polymer, would be an ideal candidate for high X-ray transparency (Sui *et al.*, 2016 ▸). Additionally, high levels of cross-linking can help to minimize water loss through such a film, providing stability against the types of polar solvents like DMSO and acetone that are commonly used for small-molecule-compound solubilization. Beyond these criteria, mechanical stability, wetting properties and manufacturability are also important considerations.

We selected an epoxy-based photoresist product series (SU-8, Kayaku Advanced Materials, formerly Microchem) as the device material because of the ability to use photolithography to manufacture microscale features at high resolution, as well as its vapor barrier properties and X-ray transparency. We developed a fabrication technique to bond three lithographically patterned layers of photoresist to form a sealed microfluidic chip with tunable layer thicknesses [Figs. 1 ▸(*a*) and 1 ▸(*b*)]. The most critical step during fabrication was the bonding step, which took advantage of a thermal bonding process using nanoimprinting (Schift, 2008 ▸). This procedure first created an intimate layer contact between the different films to facilitate uniform bonding throughout the device while minimizing the potential for feature deformation. A sacrificial layer facilitated release of the photoresist structures from the silicon substrate after fabrication. The total thickness of the photoresist material present in the X-ray beam during diffraction data collection was approximately 36 µm and has the potential to be further reduced, as required, to minimize the interference of the device material with the X-ray analysis.

The layout of our device was chosen to facilitate convenient solution dispensing both manually and by automation. Each channel was designed for sample loading by capillary action with dimensions of 6.75 mm in length, 0.4 mm in width and 0.14 mm in height, resulting an approximate volume of 400 nl. The distance between two inlets of adjacent parallel channels or co-linear channels was set using the well-center intervals of a standard Corning 1536-well plate, facilitating compatibility with liquid-handling robotics [Fig. 1 ▸(*c*)]. Furthermore, a relatively large 1 mm inlet diameter allows for a degree of positioning error and/or efficient manual pipetting. The channel cross-sectional area combined with the hydrophilic nature of the cross-linked photoresist allowed for spontaneous filling of the channels via capillary action, while radially distributed small circles at the inlet and outlet reduced the spread of excess solution and helped to anchor epoxy adhesive when sealing the channel. Numbering near the inlets allows for easy identification of individual channels during dispensing, while numbering along the channels assists during synchrotron data collection. Two notches were included at the outlet end of each channel to help retain crystals within the channel [Fig. 4 ▸(*a*), insets]. A set of fiducial markers enabled precise alignment of the chip in the 3D-printed frames and cassettes, as is necessary for automated operations [Figs. 1 ▸(*c*) and 1 ▸(*d*)]. The 3D-printed frames and cassettes secured chips in place during storage, shipping and data collection and allowed for accurate positioning in an automation scenario [Figs. 1 ▸(*d*) and 1 ▸(*e*)].

Our first consideration in testing these devices was their ability to protect protein crystals against dehydration. We investigated the rate of water loss from our devices visually using optical microscopy. Image analysis of channels filled with an aqueous dye solution demonstrated only ∼10% volumetric water loss over a 6 h period under ambient laboratory conditions [Figs. 2 ▸(*a*) and 2 ▸(*c*)]. Notably, we were unable to detect significant water loss over 24 h when the device was stored in a humid environment [Figs. 2 ▸(*b*) and 2 ▸(*c*)]. These results suggest that our device should sufficiently protect samples from dehydration during the time needed for X-ray data collection in the ambient environment of a synchrotron beamline workstation, and allows for long-time sample storage under controlled humidity.

Design considerations related to the quality of the X-ray diffraction data that can be obtained from our device must address both attenuation of the X-ray signal and background scattering from the device materials. Attenuation of the X-ray signal would be expected to be similar for all hydrocarbon-based polymer materials. However, different polymers can have dramatically different levels of background scattering due to molecular ordering in the films (Sui & Perry, 2017 ▸; Greaves & Manz, 2005 ▸). We compared the integrated background scatter for our epoxy-based photoresist material with cyclic olefin copolymer (COC), which is a widely used material in X-ray-compatible devices (Perry *et al.*, 2013 ▸; Denz *et al.*, 2018 ▸; Nunes *et al.*, 2010 ▸) (Fig. 3 ▸). For samples with the same thicknesses, spin-coated photoresist SU-8 showed a lower background scattering signal compared with COC in resolution ranges relevant to crystallography experiments. We hypothesize that this difference is a result of the way in which COC and photoresist are processed: UV cross-linking of photoresist allows for the creation of a homogeneous isotropic film; however, the types of melt extrusion processes used to produce films of COC result in significant alignment of the polymer chains, which increases the intensity of the observed background signal.

### 3.2. Ligand and crystal loading   

Crystal structures of proteins bound to small-molecule compounds can be obtained by the techniques of either co-crystallization or soaking (Hassell *et al.*, 2007 ▸). The latter strategy is more amenable to a higher-throughput workflow but requires significant manual manipulations and also suffers from the drawback that protein crystals are exposed to the organic solvents like DMSO traditionally used to solubilize the small-molecule compounds. Increasing the molar concentration of small-molecule compounds can allow for very weak binding events to be detected in soaking experiments, depending on the tolerance of the crystal system to DMSO (Wang *et al.*, 2016 ▸; Hulme & Trevethick, 2010 ▸). To address this we established a protocol, inspired by previous ligand pre-coating protocols (Gelin *et al.*, 2015 ▸), whereby the channels in our device were coated with small-molecule compounds prior to crystal addition. This was achieved by dispensing small-molecule compounds dissolved in DMSO into the channel, with subsequent incubation at room temperature for at least 48 h to allow the DMSO to evaporate from the open inlets, leaving a compound-coated channel (CCC). Crystals, in their crystallization mother liquor, were then added to the CCC and the inlets were sealed with epoxy. The small-molecule compounds were re-solubilized in the crystallization mother liquor and diffused into the protein crystal structure, enabling protein–ligand binding events [Fig. 4 ▸(*a*)].

To test the ability of the chip to be used in conjunction with existing laboratory automation, we used a mosquito liquid-handling robot (SPT Labtech) (Jenkins & Cook, 2004 ▸) to deliver compounds across a chip. The mosquito liquid-handling robot uses positive-displacement-based pipetting, providing precise and repeatable nanolitre pipetting in a flexible programmable fashion. This enabled the addition of unique, or replicate, compounds that solubilized in 100% DMSO into each of the nine channel inlets on a chip. The standardized design of the chip enabled custom scripts to be created efficiently. Furthermore, the timing independence between compound coating and crystal addition allowed libraries of CCC to be prepared prior to crystal growth. Although we have proved the device compatibility with automated liquid handling, we should clarify that all the reagents and crystals used for actual synchrotron-based data collection in this paper were loaded by manual pipetting owing to the timelines along which these developments took place.

Manipulation of protein crystals to achieve both efficient transfer and minimal physical damage was a key factor in the channel design. Crystal transfer, with negligible damage to most crystal systems, was undertaken by hand or robot pipetting and did not require any special considerations. We chose, or diluted, crystallization drops such that 0.4 µl of solution contained approximately 5–10 crystals, and placed the solution over the inlet to enable capillary action to move the crystals efficiently into the channel. The advantage of having multiple crystals in a channel was to enable data redundancy, for either merging for completeness or independent validation of weak density. We did not visually observe physical damage even to large plate-like protein crystals after the transfer. The size of crystals added to the channel was not strictly controlled but was limited by the 140 µm channel height, and we found that relatively large crystals approaching 100 µm (*i.e.* crystals that filled a significant portion of the channel) were most suitable for both visual identification and data quality. Importantly, we did not experience noticeable clogging issues during crystal filling, probably because of the strong capillary action. Crystals of Gal-3 were rod like and grew routinely to a maximum dimension of 60 µm. Smaller (∼20 µm), more radiation-sensitive crystal systems, such as protein tyrosine phosphatase 1b and tankyrase, did not always allow for sufficient diffraction data to enable electron density map calculation from single crystals, and often required merging of data sets from multiple crystals within a given channel. Other tests with proprietary systems that produce crystals larger than 50 µm routinely resulted in diffraction data of lower quality than could be obtained from cryo-protected frozen crystals, but of sufficient quality to generate electron density maps demonstrating compound binding (data not shown). These initial observations and data confirmed that room-temperature data collection, for most crystal systems, optimized for speed of collection to generate electron density maps is sufficient to confirm binding events. The impact of temperature and data collection parameter changes was not evaluated in this work and will be the focus of future analyses.

After the crystals and mother liquor were loaded into the channels, the sealing step with epoxy adhesives was routinely completed within 5 min. Once secured into 3D-printed frames and cassettes, the devices were shipped in a humid environment at ambient temperature. When properly sealed and packaged, *i.e.* Styrofoam shipping boxes and wet tissues were used to minimize potential effects of mechanical jarring and humidity changes, the chip assemblies were found to ship successfully without exception. We observed no visible physical damage or excessive movement of crystals or drying of solution from the channels. The chips could also be stored for more than a week, prior to data collection, with no evident degradation.

### 3.3. X-ray diffraction data collection   

The attachment of a magnetic chip holder pin base, which has same outside form factor as a standard magnetic pin base, to the mounting frame assembly makes the device immediately compatible with most crystallographic goniostats [Fig. 5 ▸(*a*)]. For data collection at the IMCA-CAT beamline at the APS Sector 17, the relatively large translational range of the Alio goniometer allowed all positions on the chip to be accessed without the need for adapters or any modifications to the standard experimental configuration. To fully access all channels, a translation range of 4.5 mm in the vertical direction and approximately 29 mm (−24 mm and +5 mm relative to a standard 18 mm mounted cryo-loop) along the spindle axis is required. Given the small X-ray beam sizes typical of modern synchrotron beamlines (1–100 µm), the ability to keep small crystals well centered throughout the rotational moves of data collection is an important factor. The mounting frame kept the chip itself rigid, while the secure connection to the magnetic base prevented the entire device from slipping or sagging during rotation. Moreover, for room-temperature data collection, the possibility of individual crystals moving within the device can be another common challenge; however, the narrow dimensions of the channels minimized crystal movement, which proved not to be a significant concern.

For large crystals, visual centering was sometimes reliable, but typically the entire channel or region of interest was rastered using a highly attenuated X-ray beam for diffraction signal at ±45° to identify crystal locations or the best-diffracting crystals [Fig. 5 ▸(*b*)]. In some cases, a full 90° of usable data could not be collected because of the radiation sensitivity of the target crystal, and thus smaller oscillations of data were collected from multiple crystals and merged afterwards to reconstruct a more complete data set. However, for screening efforts aimed solely at identifying positive compound binding events, multiple larger wedges of data at lower resolution were found to be sufficient. Currently, our data suggest that the data-quality criteria for structure determination are not necessarily applicable to the more qualitative positive identification of extra electron density from molecular replacement phasing in the context of compound-screening experiments.

In the case of data collection on beamlines 7-1 and 12-2 at the SSRL, the ability to quickly scan a chip to identify crystals for subsequent data collection was facilitated by incorporating specific information related to the design of our chips into computer code to allow for semi-automated alignment. By simple user interactions to align the edge of the chip and the location of the ends of the channels, we were able to automatically center our devices and generate the channel outline, perform low-dose rastering for adjacent channels to locate crystal positions, and collect X-ray diffraction data [Figs. 5 ▸(*d*)–5 ▸(*f*)]. Each channel could be scanned in 5 min. This automated channel-locating capability would enable high-hit-rate whole-device scanning, as well as high-throughput data collection.

### 3.4. Primary screening by crystallography   

**Figure 6:**
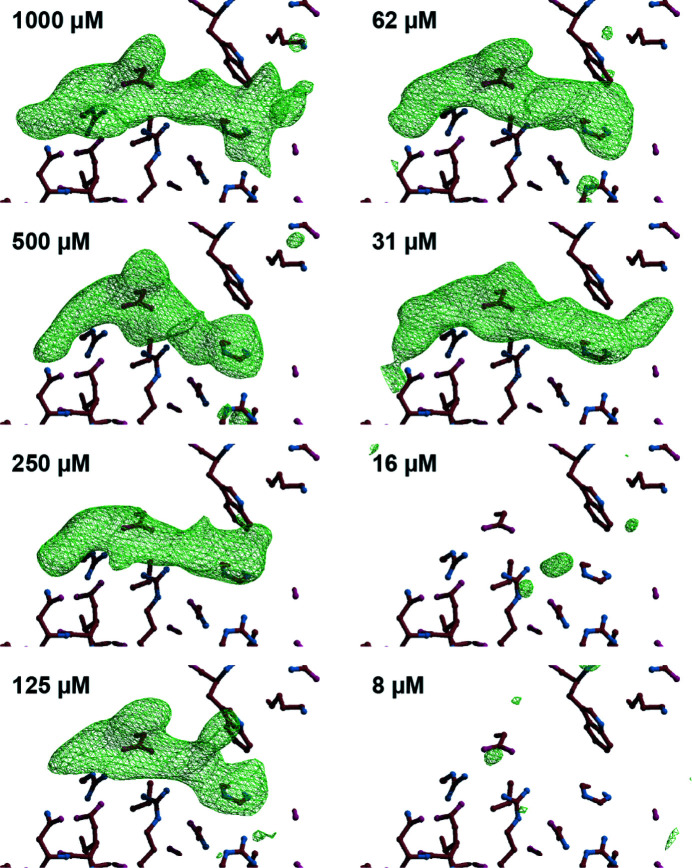
*F*_o_–*F*_c_ electron density maps for Gal-3, showing the compound density difference resulting from soaking with different concentrations of compound. Each panel reflects representative data from a single crystal soaked at the indicated compound concentration, contoured at 3σ, and the nearby Trp181.

We used Gal-3 with the TD139 compound (*K*_d_ = 14 n*M*) (Delaine *et al.*, 2016 ▸) as a test system [Fig. 5 ▸(*c*)]. A 1 m*M* stock solution of the TD139 compound was diluted twofold across eight channels in a single chip, resulting in a minimum compound concentration of 8 µ*M*. Crystals of apo-Gal-3 were then added to the channels for data collection. The individual data sets were analyzed without user intervention using a proprietary software pipeline (*SnowPipe*) based on GlobalPhasing’s *Pipedream* software (Smart *et al.*, 2012 ▸). This enabled evaluation of electron density within 60 min of data collection in a web-based display. The channels with compound concentrations above 31 µ*M* all showed clear evidence of compound binding. However, at lower compound concentrations there was no objective evidence of binding, as judged by positive difference electron density (Fig. 6 ▸). Importantly, merging of multiple data sets to iteratively improve resolution, completeness and redundancy did not change the qualitative binary assessment of compound binding (data not shown). These results suggest that primary screen data to identify small-molecule-compound binding could be achieved without the need for high-quality data or full structure determination.

To test the CCC workflow with mixtures of small-molecule compounds, we then evaluated whether the compound would still bind in the presence of 50 randomly chosen compounds. A 1 m*M* stock concentration of TD139 was dissolved with 50 compounds at 1 m*M*, and channels were coated with this mixture. Data sets were collected to 3 Å and the resulting density was consistent with TD139 binding (data not shown).

We then set about testing the throughput of our screening workflow. We evaluated the loading and data collection time needed for ten chips (90 channels). The first step of adding compound mixtures dissolved in DMSO to the capillary channels and subsequent evaporation of DMSO was tested with a random project library (not expected to bind to trypsin) of small-molecule compounds in mixtures of eight (the final concentration of each one being 6.25 µ*M*) for a total of 720 small-molecule compounds. Individual compound properties can affect the integrity of this step, and we found that approximately 10% of channels were blocked as a result of compound coagulation, an impact that can be mitigated by a prescreening step. The time to manually load 90 channels with compounds was approximately 1 h. All remaining channels were found to be DMSO free after 72 h by visual inspection. Crystals of trypsin were then added manually to each channel before sealing of the inlets, which took approximately 3 h in total. About 10% of channels did not have crystals present. This sample-handling difficulty could be addressed by more judicious source crystallization drop selections. At this point the experiment was ready to be shipped to the synchrotron for data collection. This process, even performed manually rather than with automated liquid-handling robots, represents a dramatic enhancement in throughput efficiency, as the process would take as long as 40 h for traditional manual crystal-soaking experiments.

**Figure 7:**
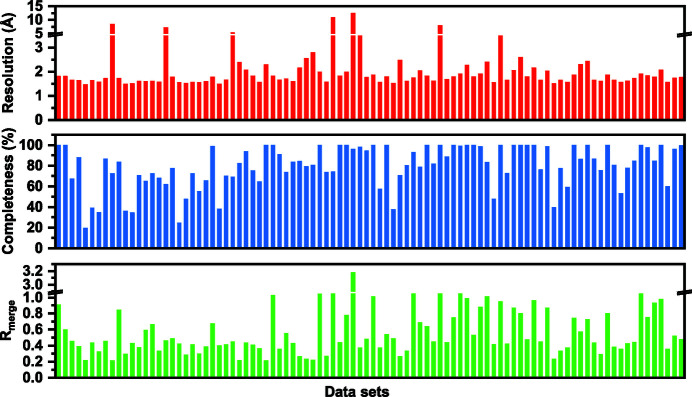
Data set statistics from trypsin screening results indicating (top) resolution, (middle) completeness and (bottom) *R*_merge_ collected from 94 individual crystals.

Data on these samples were then collected at the IMCA-CAT beamline at the APS. All samples were manually mounted and centered, and data were collected for a total of 153 crystals. Data sets were collected for 70 channels over a 14 h shift, with two channels not generating sufficient quality data for interpretable electron density maps. Fig. 7 ▸ summarizes key crystallographic parameters (completeness, resolution and *R*_merge_) obtained from 94 individual crystals successfully processed in our automated workflow; the remaining data sets failed because of sub-optimal centering and were rescued by initiating data processing when crystals moved into the beam. The average resolution across the 94 data sets was 1.8 Å, with an average completeness of >95% (space group *P*3_1_21). Thus, for a well diffracting crystal system, automated electron density maps can be efficiently evaluated for small-molecule-compound binding events from CCC-based screening.

## 4. Discussion and conclusion   

We have created a microfluidic strategy to facilitate the primary screening of small-molecule compounds via *in situ* room-temperature X-ray crystallography as part of a drug-discovery program. The microfluidic capillary channel devices provide excellent sample stability and enable the collection of high-quality X-ray diffraction data. 3D-printed frames and cassettes provide additional mechanical reliability and compatibility for automated device manipulations. We have demonstrated a compound screening workflow that reduces the time for sample preparation prior to synchrotron-based data collection and is compatible with current industrial processes. We also validated the reproducibility of the compound evaporation and data collection of the devices by obtaining high-resolution compound–protein complex structures from various projects including trypsin and Gal-3 crystals.

We established the prerequisites for CCC-based screening as being (i) protein crystals of appropriate maximum dimensions (∼100 µm for current channel dimensions), (ii) target compounds that evaporate as powders (as channels can be blocked by coagulated compounds) and (iii) project strategies that are tolerant of false negatives. Further investigation as to which X-ray diffraction data quality parameters are most critical to minimizing false-negative results will be needed. The soaking methodology is dependent on appropriately soluble compounds and is occasionally limited by the crystal structure occluding binding sites (Danley, 2006 ▸). The current nine-channel device configuration also requires goniometers with relatively large translational capabilities, although the device geometry can easily be adapted to beamlines with smaller translational capabilities.

**Figure 8:**
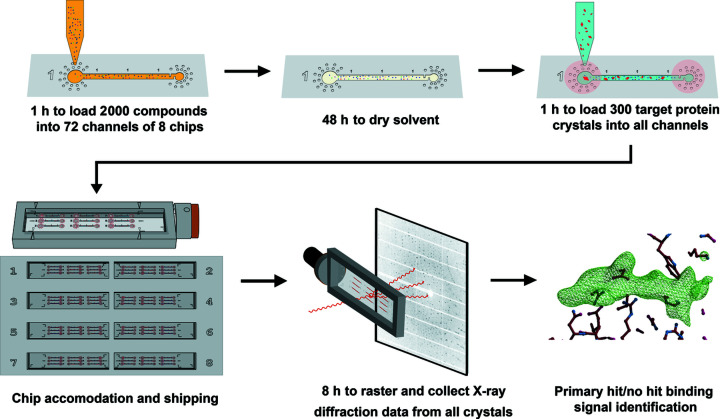
Overview of the envisioned compound screening workflow and the corresponding timeline based on full automation. From loading compound to resolving structure, use of our microfluidic platform significantly improved the efficiency of the compound-screening process.

We envision that the process efficiency will be further improved with full liquid-handling automation, compatibility with existing sample automounters and minor beamline adaptations. An optimized synchrotron data collection process for the device can be envisaged to consist of robot-assisted sample mounting onto the goniostat, followed by the centering of the chip using visual recognition software for the fiduciary marks or edge detection, X-ray rastering of each channel, centering of each crystal, and automated data collection. The data are compatible with structure determination software pipelines that are routinely used to efficiently evaluate and present electron density maps [*e.g*. *SnowPipe* (used here) or *PanDDA* (Pearce *et al.*, 2017 ▸)]. The automated workflow is presented in Fig. 8 ▸ with timelines that reflect our experience with the CCC chips. A compound library consisting of 2000 compounds in mixtures can be screened in less than a day of beam time. This report paves the way for adopting a microfluidic strategy as an advanced sample delivery method in synchrotron sources to enable the discovery of novel small-molecule binding events.

## Supplementary Material

Supplemenatgry Video 1. DOI: 10.1107/S1600576721004155/te5077sup1.mp4

Supplementary Video 2: demonstrating automated device alignment and screening at the SSRL. DOI: 10.1107/S1600576721004155/te5077sup2.mov
